# Empirical comparison of univariate and multivariate meta‐analyses in Cochrane Pregnancy and Childbirth reviews with multiple binary outcomes

**DOI:** 10.1002/jrsm.1353

**Published:** 2019-08-12

**Authors:** Malcolm J. Price, Helen A. Blake, Sara Kenyon, Ian R. White, Dan Jackson, Jamie J. Kirkham, James P. Neilson, Jonathan J. Deeks, Richard D. Riley

**Affiliations:** ^1^ Institute of Applied Health Research University of Birmingham Birmingham UK; ^2^ NIHR Birmingham Biomedical Research Centre University Hospitals Birmingham NHS Foundation Trust and University of Birmingham Birmingham UK; ^3^ Department of Medical Statistics London School of Hygiene and Tropical Medicine London UK; ^4^ Department of Health Services Research and Policy London School of Hygiene and Tropical Medicine London UK; ^5^ MRC Clinical Trials Unit University College London London UK; ^6^ Statistical Innovation Group AstraZeneca Cambridge UK; ^7^ Department of Biostatistics University of Liverpool Liverpool UK; ^8^ Cochrane Pregnancy & Childbirth Group, Centre for Women's Health Research University of Liverpool Liverpool UK; ^9^ Centre for Prognosis Research Research Institute for Primary Care & Health Sciences Keele University UK

**Keywords:** comparison, evidence synthesis, multivariate meta‐analysis, univariate meta‐analysis

## Abstract

**Background:**

Multivariate meta‐analysis (MVMA) jointly synthesizes effects for multiple correlated outcomes. The MVMA model is potentially more difficult and time‐consuming to apply than univariate models, so if its use makes little difference to parameter estimates, it could be argued that it is redundant.

**Methods:**

We assessed the applicability and impact of MVMA in Cochrane Pregnancy and Childbirth (CPCB) systematic reviews. We applied MVMA to CPCB reviews published between 2011 and 2013 with two or more binary outcomes with at least three studies and compared findings with results of univariate meta‐analyses. Univariate random effects meta‐analysis models were fitted using restricted maximum likelihood estimation (REML).

**Results:**

Eighty CPCB reviews were published. MVMA could not be applied in 70 of these reviews. MVMA was not feasible in three of the remaining 10 reviews because the appropriate models failed to converge. Estimates from MVMA agreed with those of univariate analyses in most of the other seven reviews. Statistical significance changed in two reviews: In one, this was due to a very small change in *P* value; in the other, the MVMA result for one outcome suggested that previous univariate results may be vulnerable to small‐study effects and that the certainty of clinical conclusions needs consideration.

**Conclusions:**

MVMA methods can be applied only in a minority of reviews of interventions in pregnancy and childbirth and can be difficult to apply because of missing correlations or lack of convergence. Nevertheless, clinical and/or statistical conclusions from MVMA may occasionally differ from those from univariate analyses.

## 1 INTRODUCTION

Meta‐analysis is an umbrella term for a suite of statistical models for synthesizing parameter estimates (eg, intervention effect estimates) from multiple studies. Each model implies a set of assumptions, such as fixed or random intervention effects.^1^ Often, a review has several outcomes of interest (such as preterm delivery and neonatal intensive care), and the effect estimates for these outcomes may be correlated within primary studies, because the same patients provide data towards them. Standard univariate meta‐analysis (UVMA) methods do not account for this correlation. In recognition of this, multivariate meta‐analysis (MVMA) models have been developed.^2^, ^3^, ^4^ Multivariate models have a number of potential advantages over univariate counterparts. They facilitate borrowing of strength across outcomes,^5^ which utilizes more information and thereby potentially reduces uncertainty and the impact of outcome reporting bias.^6^ They also facilitate estimation of joint confidence and prediction regions^4^, ^7^ and allow appropriate confidence intervals (CIs) to be calculated for functions of summary estimates for multiple correlated outcomes.^8^

Empirical evidence of the impact of MVMA on results and conclusions is limited. The MVMA model is potentially more difficult and time‐consuming to apply than univariate models, so if its use makes little difference to parameter estimates, it could be argued that it is redundant. Trikalinos et al^9^ reported results of a systematic investigation of the difference in results across all reviews published by Cochrane in the first quarter of 2012 for which the MVMA model was readily applicable. They concluded that the difference between univariate and multivariate results was generally small and usually clinical conclusions did not change. This finding concords with results from many of the example datasets analyzed in methodological papers.^10^, ^11^ However, there are also many examples that suggest the impact of MVMA can be large,^5^, ^6^ especially when there are (selectively) missing outcomes.^12^, ^13^

The literature suggests that the difference between univariate and multivariate results tends to be greater in circumstances where the outcomes are highly correlated and some studies do not report all outcomes (ie, there is missing outcome data). Such situations often occur in reviews performed by the Cochrane Pregnancy and Childbirth (CPCB) Group, which routinely examines multiple outcomes for both the mother and baby,^14^ meaning that MVMA is more likely to have an impact in this clinical area. In this paper, we use both univariate and MVMA models to analyze aggregate data reported in CPCB reviews. The purpose of the paper is threefold: first, to identify how often the multivariate model is reasonably applicable in CPCB reviews; second, to determine how often, and to what degree, the use of the multivariate model leads to different statistical results and conclusions than those obtained from the standard univariate model; and third, to highlight any circumstances where reported clinical conclusions in these reviews should potentially be reconsidered in light of results from multivariate models.

## 2 METHODS

### 2.1 Inclusion criteria

We screened the CPCB database^15^ to identify all reviews, new or updated, published between January 2011 and February 2013. Only reviews of interventions were considered. If a review was published and updated during this period, then we only considered the most recent version.

Each review was screened by M.J.P. for whether it contained eligible outcomes. We considered an outcome to be eligible if it was a binary primary outcome and was reported by three or more studies. We considered a review to be potentially suitable for MVMA if it had two eligible maternal outcomes or two eligible neonatal outcomes. Some reviews reported multiple intervention contrasts (eg, results comparing intervention A versus B and B versus C). If more than one contrast would be eligible, we considered only the first intervention contrast listed in the review that fulfils these criteria. We then choose either multiple maternal outcomes or multiple neonatal outcomes on the basis of whichever was reported first. Then, all binary primary outcomes of this type reported by at least three studies were analyzed. Subgroup analysis results were not included. We limited the analysis to primary outcomes to keep the dataset manageable and to maintain focus on the most clinically relevant outcomes.

For each included outcome in each review, the outcome description, intervention description, and the number of events and number of patients at follow‐up (ie, a standard two‐by‐two data table) in each arm were extracted. Data extraction was performed by two statisticians independently (M.J.P. and H.B.). The datasets were compared, and any differences reconciled upon discussion with a third reviewer as necessary. Data and outcomes where discussed with clinical collaborators before analyses were undertaken.

### 2.2 Evidence synthesis methods

For each review that met the inclusion criteria, and for each outcome identified, we derived the log odds ratio (OR) estimates and their variances for each primary study. If no events occurred in either arm, then the corresponding outcome was treated as missing in that study. This is in keeping with the usual approach to UVMA in which studies with no events in either arm are given zero weight.^16^ If this was the case for all outcomes (to be analyzed) in a study, then the study was excluded from the analysis. If 0% or 100% of patients in just one arm had events, then 0.5 was added to each cell in order to derive the log OR and its standard error.^16^, ^17^

We then applied, to each meta‐analysis dataset and to each outcome separately, the standard univariate random effects meta‐analysis model fitted using restricted maximum likelihood estimation (REML) (Appendix S1).^1^, ^18^, ^19^ The random effects model was used to account for heterogeneity, which was expected in most CPCB reviews.^14^ Meta‐analysis was performed on the log OR scale, and the summary results were expressed as ORs, with 95% CIs.

If within‐study correlations between treatment effects on outcomes were calculable, we attempted to fit a fully hierarchical MVMA (here, bivariate) random effects model (Appendix S2) to each pair of outcomes in each review. As we only had access to summary level data reported in journal articles, this was only possible if the outcomes were structurally related, as follows. If the outcomes were mutually exclusive, such as vaginal birth and cesarean section, then within‐study correlations were estimated using the method of Trikalinos and Olkin (see their equation A3 in their appendix A).^20^ If one outcome was a subset of the other (eg, cesarean section is a subset of operative birth), the method shown by Trikalinos and Olkin and derived by Wei and Higgins (see their equation 10)^21^, ^22^ was used. In both cases, correlations in the treatment effect estimates for each pair of outcomes are induced because of the binary outcomes being negatively correlated for mutually exclusive outcomes and positively correlated for subset outcomes. In both papers, analytical solutions for deriving these within‐study correlations are given, which require the meta‐analyst to input the number of participants and number of events in each trial arm for each outcome. If neither of these two methods to calculate within‐study correlations were applicable, we applied the “Riley model,” the alternative MVMA model that does not require within‐study correlations as it models an amalgamation of the within‐study and between‐study correlations (see Appendix S3).^3^ Finally, for each review containing more than two eligible outcomes, an MVMA model was fitted to *all* outcomes if possible, either using the fully hierarchical approach or using the Riley model (eg, a trivariate model was fitted if three outcomes were eligible). All UVMA and MVMA models were estimated in STATA version 14 using restricted maximum likelihood via the mvmeta module.^23^, ^24^ Standard errors for the summary estimates account for uncertainty in the between‐study variance and covariance matrix estimates.^23^ Computational methods including criteria for lack of convergence of the Riley model are outlined in Appendix S4.

Results from UVMA and MVMAs were compared by inspection of their summary estimates and CIs and in particular the impact on the clinical and/or statistical conclusions that would be drawn.

## 3 RESULTS

### 3.1 Identification of reviews

Our search identified 80 CPCB reviews published between January 2011 and February 2013. Of these, 27 reviews (34%) included at least one eligible outcome as defined above, of which 10 included at least two primary binary maternal or child outcomes for the same intervention contrast, and hence were potentially suitable for MVMA. A more detailed breakdown of the results at each stage of the selection process is given in Table 1.

**Table 1:** Nested criterion for inclusion (number remaining reports the number of reviews remaining after this criterion has been assessed)

Criteria	Number of Reviews Meeting Criteria
Most recent Cochrane Pregnancy and Childbirth reviews published between January 2011 and February 2013	80
Reviews contained at least three studies in total	46
At least one outcome was reported by three or more studies	31
At least one binary outcome was reported by three or more studies	29
At least one binary primary outcome was reported by three or more studies	27
At least two (including 1+ primary) binary outcomes, both maternal or both neonatal, were reported by three or more studies	18
At least two binary primary outcomes, both maternal or both neonatal, were reported by three or more (not necessarily the same) studies	10
MVMA models converged for at least one pair of outcomes	7

*Note*. Twenty‐one studies included a contrast with at least two mother or two neonatal binary (either primary or secondary) outcomes reported by three or more (not necessarily the same) studies.

Abbreviation: MVMA, multivariate meta‐analysis.

### 3.2 Results from analyzed reviews

Of the 10 eligible reviews, two, three, and four eligible outcomes were identified in seven, two, and one of the reviews, respectively. Within‐study correlations were calculable in four of the reviews (two contained outcomes that were mutually exclusive, and the other two contained outcomes with a subset relationship). In the other six reviews, only the Riley model could be considered: In three of these, it did not converge for any pair of outcome using any of the methods described in Appendix S4. Hence, multivariate results were only available for seven of the 10 reviews. These seven reviews are now discussed in turn, and results are presented in Table 2:

Review 1 Cardiotocography versus intermittent auscultation of fetal heart on admission to labor ward for assessment of fetal wellbeing^25^

**Table 2:** Univariate and bivariate summary odds ratio estimates, 95% confidence intervals, and between‐study standard deviation estimates for the pooled log odds ratio for each outcome in each review

Summary OR Estimates (95% CI)									
Outcome (Number of Studies)	Univariate^a^	Bivariate (1,2)^a^	Bivariate (1,3)^a^	Bivariate (2,3)^a^	Bivariate (1,4)^a^	Bivariate (2,4)^a^	Bivariate (3,4)^a^	Trivariate (1,2,4)^a^	Trivariate (1,3,4)^a^
Review 1: Cardiotocography versus intermittent auscultation of fetal heart on admission to labor ward for assessment of fetal wellbeing^25^									
(1) Caesarean birth (4)	1.21 (1.00‐1.46) 0.00	1.23 (0.99‐1.52) 0.08	–	–	–	–	–	–	–
(2) Instrumental vaginal birth (4)	1.12 (0.95‐1.31) 0.09	1.12 (0.96‐1.31) 0.09	–	–	–	–	–	–	–
Review 2: Cervical stitch (cerclage) for preventing preterm birth in singleton pregnancy^26^									
(1) All perinatal losses (8)	0.77 (0.59‐1.02) 0.00	0.77 (0.59‐1.02) 0.00	0.77 (0.59‐1.02) 0.00	–	–	–	–	–	–
(2) Serious neonatal morbidity (4)	0.94 (0.59‐1.49) 0.00	0.94 (0.60‐1.48) 0.04	–	0.91 (0.58‐1.42) 0.03	–	–	–	–	–
(3) Perinatal deaths or serious neonatal morbidity (4)	0.78 (0.53‐1.14) 0.06	–	0.77 (0.57‐1.04) 0.00	0.81 (0.53‐1.25) 0.22	–	–	–	–	–
Review 3: Hypnosis for pain management during labor and childbirth^27^									
(1) Use of pharmacological pain relief (6)	0.35 (0.14‐0.87) 0.98	0.37 (0.15‐0.89) 0.93	–	–	–	–	–	–	–
(2) Spontaneous vaginal birth (4)	2.91 (0.63‐13.5) 1.23	3.23 (0.72‐14.6) 1.10	–	–	–	–	–	–	–
Review 4: Intracutaneous or subcutaneous sterile water injection compared with blinded controls for pain management^28^									
(1) Assisted vaginal birth (6)	1.28 (0.58‐2.83) 0.19	1.16 (0.51‐2.60) 0.34	–	–	–	–	–	–	–
(2) Caesarean section (7)	0.55 (0.29‐1.05) 0.00	0.58 (0.28‐1.19) 0.28	–	–	–	–	–	–	–
Review 5: Tocolytics for preterm premature rupture of membranes^29^									
(1) Perinatal mortality (7)	1.71 (0.78‐3.75) 0.00	1.72 (0.78‐3.76) 0.00	–	–	–	–	–	–	–
(2) Neonatal death (7)	1.71 (0.75‐3.89) 0.00	1.72 (0.78‐3.77) 0.00	–	–	–	–	–	–	–
Review 6: Inhaled analgesia for pain management in labor^30^									
(1) Satisfaction with pain relief (4)	0.63 (0.38‐1.06) 0.00	0.63 (0.37‐1.07) 0.00	FC	–	–	–	–	–	–
(2) Assisted vaginal birth (5)	0.58 (0.26‐1.29) 0.64	0.59 (0.25‐1.42) 0.65	–	0.58 (0.25‐1.38) 0.66	–	–	–	–	–
(3) Vomiting (3)	2.19 (0.66‐7.24) 0.00	–	FC	2.55 (0.41‐16.0) 0.01	–	–	–	–	–
Review 7: Interventions for preventing nausea and vomiting in women undergoing regional anesthesia for cesarean section^31^									
(1) Nausea‐intraoperative (8)	0.39 (0.20‐0.76) 0.72	0.38 (0.19‐0.76) 0.76	FC	–	0.39 (0.19‐0.78) 0.77	–	–	0.38 (0.19‐0.78) 0.85	0.38 (0.16‐0.91) 0.98
(2) Vomiting‐intraoperative (7)	0.41 (0.17‐0.96) 0.85	0.36 (0.14‐0.94) 0.99	–	0.56 (0.25‐1.23) 0.81	–	0.41 (0.18‐0.98) 0.86	–	0.37 (0.15‐0.92) 0.86	
(3) Nausea‐postoperative (4)	0.24 (0.06‐0.87) 1.16	–	FC	0.24 (0.10‐0.58) 0.76	–	–	0.24 (0.06‐0.86) 1.15	–	0.31 (0.13‐0.74) 0.72
(4) Vomiting‐postoperative (5)	0.30 (0.18‐0.48) 0.00	–	–	–	0.30 (0.19‐0.48) 0.00	0.29 (0.17‐0.50) 0.00	0.30 (0.18‐0.48) 0.00	0.37 (0.21‐0.63) 0.23	0.29 (0.13‐0.64) 0.83

*Note*. All remaining trivariate and quadvariate models failed to converge.

Abbreviations: CI, confidence interval; FC, failed to converge; OR, odds ratio.

a Summary estimates of the OR (95% CIs) between‐study standard deviation estimates for the pooled log OR.

This review compares admission cardiotocography versus intermittent auscultation for outcomes of (1) cesarean section and (2) instrumental vaginal birth. These two outcomes are mutually exclusive, and therefore, within‐study correlations could be derived (range from lowest to highest within‐study correlation −0.02 to −0.10). The comparison includes four trials all reporting both outcomes. The between‐study correlation is estimated to be +1, and the summary intervention effect estimates and CIs are almost identical for bivariate and univariate models (Table 2). In the univariate model, the CI for the intervention effect on cesarean section excludes the null value (summary OR: 1.21; 95% CI, 1.00‐1.46) whereas in the bivariate model, it includes it (summary OR: 1.23; 95% CI, 0.99‐1.52). This slight change should not affect the clinical conclusions for this outcome, although statistical significance at the conventional 5% level is affected, because of the *P* value in the MVMA being >0.05.

Review 2 Cervical stitch (cerclage) for preventing preterm birth in singleton pregnancy (review)^26^

This review compares cerclage versus no cerclage for outcomes of (1) all perinatal losses, (2) serious neonatal morbidity, and (3) the composite outcome of perinatal deaths and serious neonatal morbidity. Outcomes 1 and 2 are subsets of outcome 3, and therefore, using the approach of Wei and Higgins,^22^ the within‐study correlations could be derived between outcomes 1 and 3 and outcomes 2 and 3 (range of within‐study correlations from lowest to highest 0.64‐1.00). This allowed use of the fully hierarchical model for these two analyses. However, outcomes 1 and 2 are not mutually exclusive, nor is one a subset of the other, and so their within‐study correlations were not obtainable. Therefore, the Riley model was implemented for a bivariate analysis of outcomes 1 and 2 and for a trivariate analysis of all three outcomes, but for the latter, it did not converge.

Results from the three univariate and bivariate analyses are shown in Table 2. Eight studies report outcome 1, and four of these report outcomes 2 and 3; thus, there is a large proportion of missing data for outcomes 2 and 3. The Riley model applied to outcomes 1 and 2 estimated the overall correlation to be about −0.3, while the fully hierarchical bivariate model gave between‐study correlation estimates of +1 and −1 for outcomes 1 and 2 and outcomes 1 and 3, respectively. Despite the very high correlations and considerable missing data, the summary meta‐analysis estimates were similar in univariate and bivariate models, and statistical/clinical conclusions remain the same. The main difference was seen in the CI for the summary intervention effect for outcome 3, which was somewhat narrower from the bivariate analysis of outcomes 1 and 3 than the univariate analysis (Table 2).

Review 3 Hypnosis for pain management during labor and childbirth^27^

This review compares self‐hypnosis or hypnotherapy versus control for outcomes of (1) use of pharmacological pain relief/anesthesia and (2) spontaneous vaginal birth. Six studies reported outcome 1, and four of these reported outcome 2. There is no structural relationship between these outcomes, so no within‐study correlations could be derived. The Riley model converged, and the univariate and bivariate estimates of the intervention effects are shown in Table 2. The overall correlation was estimated as −0.44. Meta‐analysis estimates and CIs are fairly similar between the univariate and multivariate models, and the latter would not alter the original statistical or clinical conclusions from the univariate analyses (Table 2).

Review 4 Intracutaneous or subcutaneous sterile water injection compared with blinded controls for pain management^28^

This review compares sterile water versus normal saline for outcomes of (1) assisted vaginal birth and (2) cesarean section. These outcomes are mutually exclusive, so within‐study correlations were derivable (range −0.07 to −0.14). Seven studies reported outcome 2, of which six reported outcome 1. The between‐study correlation was estimated to be −1. The CIs for outcome 1 were wider for the bivariate model than for the univariate because the between‐study standard deviation was estimated to be about twice as large. Estimates for outcome 2 were very similar in bivariate and univariate models, although the between‐study standard deviation was zero in the univariate model and 0.28 in the bivariate model. Statistical and clinical conclusions would likely remain unchanged between univariate and bivariate results (Table 2).

Review 5 Tocolytics for preterm premature rupture of membranes (review)^29^

This review compares tocolytic versus no tocolytic for outcomes of (1) perinatal mortality and (2) neonatal death. Outcome 2 is a subset of outcome 1, and so within‐study correlations were derivable. The same seven studies provide data on both outcomes. The number of events for outcomes 2 and 1 in both arms is the same in six of the seven trials generating within‐study correlations of +1. The between‐study correlation is estimated to be almost 1. Univariate and bivariate results are almost identical, and thus, statistical and clinical conclusions remain unchanged (Table 2).

Review 6 Inhaled analgesia for pain management in labor (review)^30^

This review compares nitric oxide versus flurane for the outcomes of (1) satisfaction with pain relief, (2) assisted vaginal birth, and (3) vomiting. Four studies reported outcome 1, these and one further study reported outcome 2, and two studies (one also reporting outcomes 1 and 2, the other just outcome 2) reported outcome 3. There is no structural relationship between these outcomes, so no within‐study correlations could be derived, and therefore, the Riley model was fitted. The model converged for the bivariate analyses of outcomes 1 and 2 and outcomes 2 and 3. Univariate and bivariate analyses gave very similar estimates and CIs for outcomes 1 and 2. For outcome 3, the summary intervention effect estimate was slightly higher and had a wider CI from the MVMA analysis. However, in all cases, clinical and statistical conclusions would remain unchanged (Table 2).

Review 7 Interventions for preventing nausea and vomiting in women undergoing regional anesthesia for cesarean section^31^

**Figure 1:**
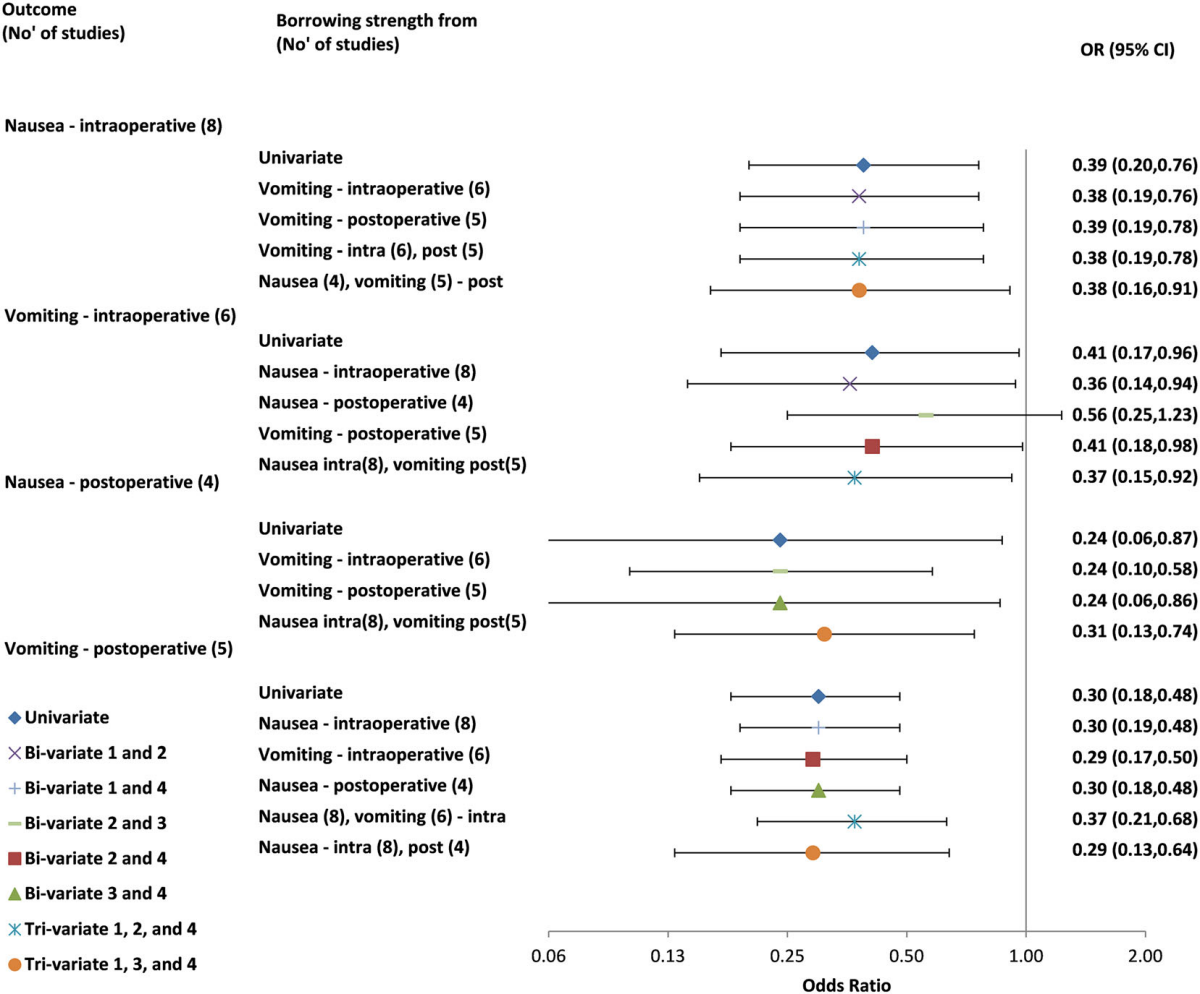
Univariate and multivariate meta‐analysis results for all outcomes from review 7: interventions for preventing nausea and vomiting in women undergoing regional anesthesia for cesarean section.^31^ CI, confidence interval; OR, odds ratio [Colour figure can be viewed at wileyonlinelibrary.com]

The contrast used compares 5‐HT3 antagonists versus placebo for outcomes of (1) intraoperative nausea, (2) intraoperative vomiting, (3) postoperative nausea, and (4) postoperative vomiting. Eight studies reported outcome 1 and seven of these outcome 2. Two of these studies and two other studies reported outcome 3. The same four studies reported outcome 4 along with a study that had reported outcomes 1 and 2. There is no structural relationship between these outcomes, so no within‐study correlations could be derived, and therefore, the Riley model was fitted. All bivariate models converged apart from the model including outcomes 1 and 3. Trivariate models including outcomes 1, 2, and 4, and 1, 3, and 4 also converged (Figure 1). The overall correlation coefficients were generally low to moderate (range −0.07 to 0.60) but high in the bivariate analysis between outcomes 2 and 3 (0.93) and between outcomes 1 and 4 in the two trivariate analyses that converged (0.85, 0.98) (0.52 in the bivariate model).

For outcomes 1 and 4, UVMA and all MVMA models give similar estimates and CIs. However, the bivariate model of outcomes 2 and 3 leads to different conclusions about the evidence of effect for outcome 2. The estimated MVMA summary OR for outcome 2 was 0.56 (95% CI, 0.25‐1.23). This OR is less extreme than the UVMA estimated OR of 0.41 (95% CI, 0.17‐0.96). Viewed on the log OR scale, the MVMA estimate is a little more precise (standard error 0.81) than the UVMA estimate (standard error 0.85). There is far less evidence to suggest a beneficial intervention effect, with the CI substantially overlapping 1.

**Figure 2:**
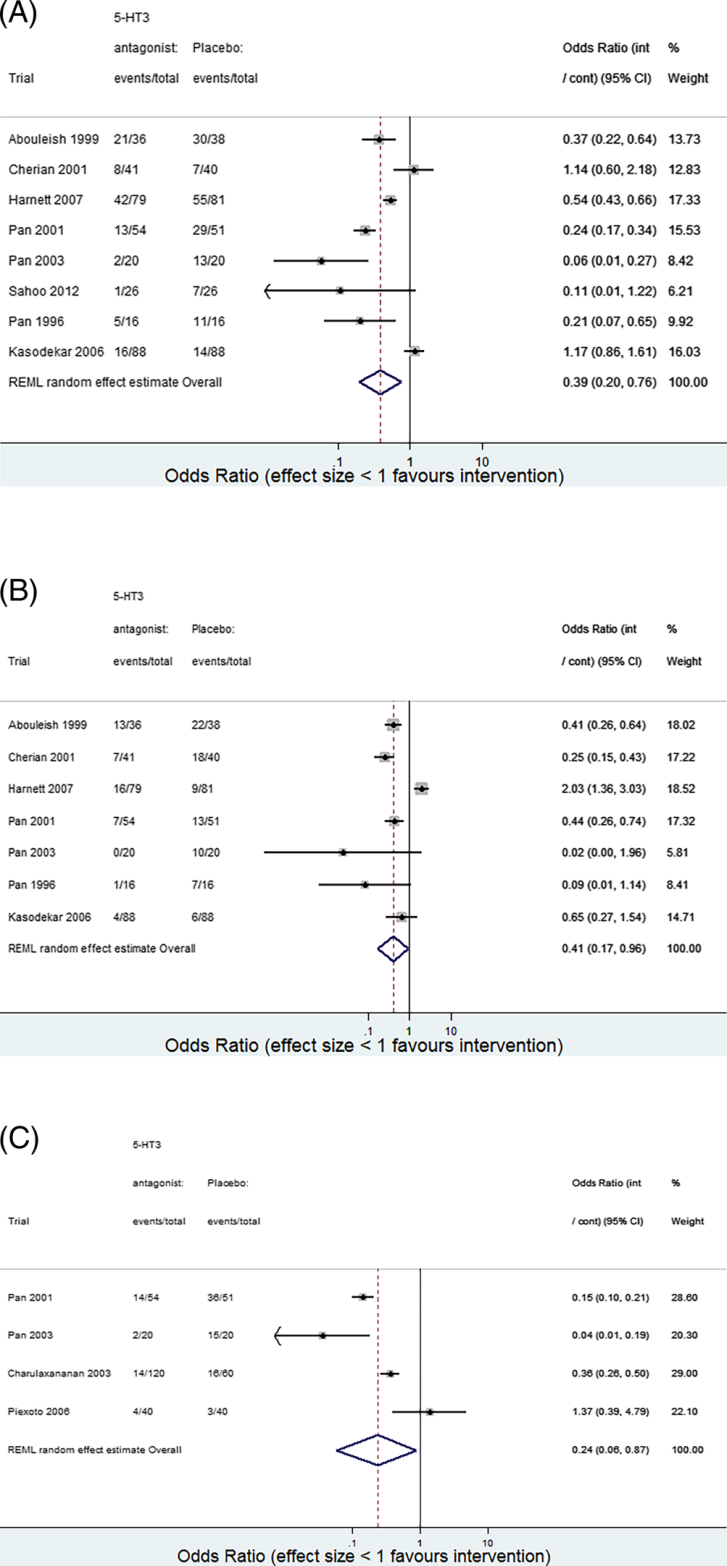
Outcome‐specific forest plots for review 7: interventions for preventing nausea and vomiting in women undergoing regional anesthesia for cesarean section.^31^ A, Outcome 1: nausea‐intraoperative. B, Outcome 2: vomiting‐intraoperative. C, Outcome 3: nausea‐postoperative. CI, confidence interval. int means intervention; arm and cont means control arm; Restricted Maximum Likelihood Estimation (REML) full trial references can be found in reference 31 [Colour figure can be viewed at wileyonlinelibrary.com]

**Figure 3:**
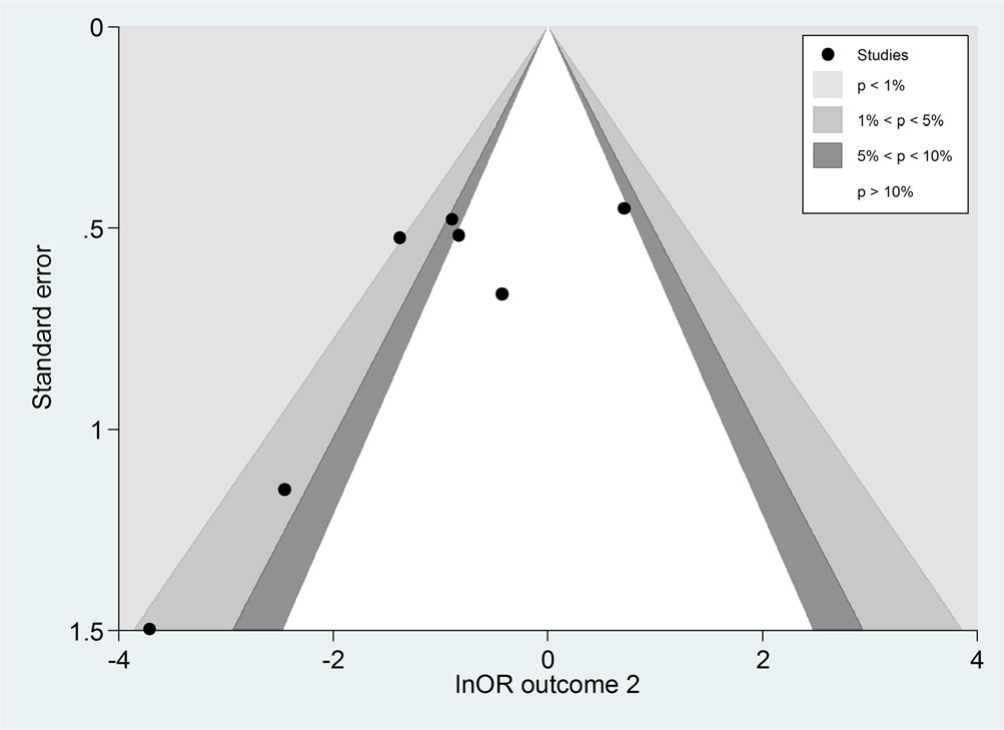
Contour‐enhanced funnel plot for examining small‐study effects in the univariate meta‐analysis for outcome 2 (vomiting‐intraoperative) in review 7: interventions for preventing nausea and vomiting in women undergoing regional anesthesia for cesarean section.^31^ OR, odds ratio [Colour figure can be viewed at wileyonlinelibrary.com]

This large shift triggered us to examine whether there was evidence of small‐study effects for outcome 2 in the UVMA.^6^ Outcome‐specific forest plots are shown in Figure 2. A contour‐enhanced funnel plot was produced, and this indeed revealed visual evidence of asymmetry for outcome 2 (Figure 3): The smaller studies tended to give more optimistic estimates of the intervention effect for outcome 2 than the larger studies.

The MVMA model including outcomes 1 and 3 with outcome 2 allowed inclusion of results from three further studies (outcome 1 provides an additional study, and outcome 3 provides an additional two studies) compared with those in the UVMA of outcome 2. However, their inclusion shifted the summary estimate for outcome 2 in opposite directions in the bivariate analyses. We therefore approximated a trivariate model of outcomes 1, 2, and 3 to ascertain whether the univariate conclusions about outcome 2 were robust or not. We fitted the Riley model with correlations fixed to the values estimated in the bivariate analyses (0.60 for outcomes 1 and 2; 0.93 for outcomes 2 and 3; and 0 for outcomes 1 and 3 where the bivariate analysis failed to converge). This gave a summary OR for outcome 2 of 0.55 (95% CI, 0.24‐1.26).

We conclude that, because of the small‐study effects and the impact of the correlation in MVMA when additionally incorporating outcomes 1 and especially 3, the results are not robust to model choice. Therefore, results from any model for this outcome should be treated with caution.^6^

## 4 DISCUSSION

In around 40% of the CPCB reviews screened, at least one outcome was reported by three or more studies, and MVMA of two or more primary maternal or child binary outcomes was prima facie possible in 13%. If the criteria were relaxed to analyze a primary and secondary outcome, this increased to about 60%. We were able to fit the MVMA model for at least one pair of outcomes in seven of the 10 contrasts considered (one contrast from each review). In four of these seven reviews, within‐study correlations could be estimated for at least one pair of outcomes. In the remaining reviews, we attempted to fit the Riley model. This model has been shown, through simulations, to produce approximately unbiased summary results, appropriate coverage, and increased precision compared with separate univariate analyses.^3^ However, it is an approximation and generally does not perform as well as a fully hierarchical multivariate model that includes within‐study correlations. Thus, if within‐study correlations are available, the fully hierarchical model is preferred. The model converged for at least one pair of outcomes in three of the six remaining reviews. Thus, convergence and estimation difficulties are common for MVMA models.

Overall, in five of the seven contrasts where an MVMA could be fitted, statistical and clinical conclusions remained unchanged. In the other two contrasts, there was at least one outcome for which results would classically be labelled as “statistically significant” at the 5% level under the univariate model but not under the multivariate model. The first was for the cesarean section outcome in a comparison of cardiotocography versus intermittent auscultation of fetal heart on admission to labor ward for assessment of fetal wellbeing (review 1). The univariate and multivariate estimates were almost identical, but in this case, the CIs for the former crossed the null value but did not for the latter. This says more about the dangers of using the concept of statistical significance than it does the use of MVMA.

The other was for the intraoperative vomiting outcome in review 7, comparing 5‐HT3 antagonists versus placebo for preventing nausea and vomiting in women undergoing regional anesthesia for cesarean section.^31^ In this review, results from the univariate model suggested good evidence of an improvement with intervention and that this effect could be quite large. However, when correlated data on the outcome of postoperative nausea were also utilized in the MVMA, results from the Riley model changed the conclusion about the effect on vomiting: Now, there was very little evidence to conclude an effect with the OR closer to 1 and wide CIs (although a strong clinically important effect could not be ruled out). Assessment of funnel plot asymmetry suggests the UVMA may be vulnerable to small‐study effect bias in outcome 2, possibly because of publication and/or outcome reporting bias. The MVMA partially corrects for bias introduced by this mechanism, by utilizing additional studies via the correlated outcome of postoperative nausea.^6^, ^32^ However, conclusions about use of the treatment are unlikely to change because of what is still a large observed central estimate for the effect together with large improvements for other outcomes.

Sometimes CIs were wider after MVMA, compared with UVMA; this is because allowing for correlation may lead to increases in the between‐study variance estimates, which then lead to wider CIs. The associated difficulties for borrowing of strength statistics when estimated between‐study variances differ between univariate and multivariate models have been discussed previously.^33^, ^34^ In such situations, borrowing of strength still occurs, but gain in precision of the pooled estimates is not observed because of the larger variance estimates and borrowing of strength statistics can be negative.

Our review has limitations. Our analysis only considered binary outcomes, and we could only fit a fully hierarchical model when within‐study correlations could be estimated directly from summary level data. However, a number of other options could have been considered. For example, a deterministic sensitivity analysis could have been performed to examine the impact of different plausible levels of correlation. Alternatively, we could have attempted to obtain individual patient data allowing within‐study correlations to be estimated for all outcomes. Finally, the correlations could have been estimated from external evidence and incorporated using a Bayesian framework perhaps using individual patient data from a subset of the trials. This could have been aided by a reparameterization of the model in terms of patient‐level correlations between outcomes (rather than contrasts) as suggested by Wei and Higgins.^22^ In addition, Bayesian methods with informative priors could have been considered when the multivariate models (either fully hierarchical or the overall correlation model) failed to converge. However, we deliberately did not consider these alternative methods as they are more complex to implement and would be harder to implement routinely within Cochrane.

A recent paper by Trikalinos et al covered a far broader set of reviews^9^ and showed that MVMA usually makes little difference. However, within their review, there were examples where univariate and MVMAs led to different summary results and conclusions. Having established this, we sought to focus on the clinical area of pregnancy and childbirth, to see if MVMA is more consistently beneficial in an area where multiple and correlated outcomes are routinely encountered. This narrower focus also allowed us to consider the results of each analysis in much more detail and discuss the results with clinicians and epidemiologists who are experts in the area.

MVMA allows joint inferences to be made for intervention effects on multiple outcomes. It is worth noting that even if multivariate and univariate summary results are identical, subsequent postestimation analyses (eg, economic models) that include intervention effect estimates for more than one of these outcomes will be incorrect if they do not allow for associations between outcomes. This is because decision and economic models require joint inferences about (functions of) the multiple outcomes, such as the probability of both outcomes 1 and 2 occurring, which requires their correlation to be accounted for.^7^, ^35^ So in such cases if multivariate synthesis is feasible, it will always be preferable to multiple univariate syntheses.

Further methodological work is required to identify scenarios where MVMA is likely to be of value. A recent paper demonstrated how the amount of borrowing of strength between outcomes can be summarized in a single statistic.^33^ However, this requires the MVMA to have been performed. A quick and easy method of determining whether MVMA is likely to be worthwhile before the analysis is performed would be of great value for researchers, many of whom are not statisticians (eg, in Cochrane). To avoid the possibility of researchers only reporting MVMA when the results move in a certain direction, the criteria for undertaking an MVMA should be prespecified in the analysis protocol. Work is needed to consider which outcomes should be included in an MVMA and to assess the sensitivity of results to this choice where necessary. Finally, improved computation methods are required to ensure MVMA models converge more often. One avenue for investigation is the use of Bayesian methods with informative priors for the heterogeneity variances.^36^

## 5 CONCLUSIONS

MVMA is a useful method in principle, as the utilization of additional information in MVMA will often lead to stronger (more precise) inference and may even, on occasion, change recommendations obtained by UVMA. However, our review shows that it is currently not easy to implement, may not converge, and often does not have a big impact on intervention effect estimates or standard errors. Our findings largely concord with a previous empirical evaluation across all Cochrane clinical groups.^9^ MVMA should thus not be routinely used in Cochrane meta‐analyses. However, it may be useful in certain situations, especially where there are missing data for some outcomes or where postestimation modelling requires intervention effect estimates for multiple correlated outcomes. Because of new MVMA results identified by our review, clinical conclusions about the effect of 5‐HT3 antagonists on intraoperative vomiting may need to be more cautious than originally thought.

## AUTHORS CONTRIBUTION

M.J.P. screened the reviews and drafted the paper. M.J.P. and H.B. extracted data and performed statistical analysis. R.D.R., D.J., I.R.W., and J.J.K. conceived the idea for the project. J.N. and S.K. provided clinical advice. All authors contributed to study design and interpretation of results and commented on drafts of the paper.

## CONFLICT OF INTEREST

The authors declare that they have no conflict of interest.

## FUNDING

The work was funded by Medical Research Council project grant MR/J013595/1. Sara Kenyon was part funded by the NIHR CLAHRC West Midlands+ initiative. Ian White was also supported by the Medical Research Council Unit (program number: MC_UU_12023/21). This paper presents independent research, and the views expressed are those of the author(s) and not necessarily those of the NHS, the NIHR, or the Department of Health.

## DATA AVAILABILITY STATEMENT

Data sharing is not applicable to this article as no new data were created or analyzed in this study.

## Supporting information

Appendix S1: Univariate random effects model

Appendix S2: Multivariate random effects model

Appendix S3: Riley overall correlation model

Appendix S4: Computational methods

## References

[1] Riley RD , Higgins JP , Deeks JJ . Interpretation of random effects meta‐analyses. BMJ. 2011;342(feb10 2):d549. 21310794 10.1136/bmj.d549

[2] Nam IS , Mengersen K , Garthwaite P . Multivariate meta‐analysis. Stat Med. 2003;22(14):2309‐2333. 12854095 10.1002/sim.1410

[3] Riley RD , Thompson JR , Abrams KR . An alternative model for bivariate random‐effects meta‐analysis when the within‐study correlations are unknown. Biostatistics. 2008;9(1):172‐186. 17626226 10.1093/biostatistics/kxm023

[4] Van Houwelingen HC , Arends LR , Stijnen T . Advanced methods in meta‐analysis: multivariate approach and meta‐regression. Stat Med. 2002;21(4):589‐624. 11836738 10.1002/sim.1040

[5] Riley RD , Abrams KR , Lambert PC , Sutton AJ , Thompson JR . An evaluation of bivariate random‐effects meta‐analysis for the joint synthesis of two correlated outcomes. Stat Med. 2007;26(1):78‐97. 16526010 10.1002/sim.2524

[6] Kirkham JJ , Riley RD , Williamson PR . A multivariate meta‐analysis approach for reducing the impact of outcome reporting bias in systematic reviews. Stat Med. 2012;31(20):2179‐2195. 22532016 10.1002/sim.5356

[7] Riley RD , Price MJ , Jackson D , et al. Multivariate meta‐analysis using individual participant data. Res Synth Methods. 2015;6(2):157‐174. 26099484 10.1002/jrsm.1129 PMC4847645

[8] Thompson JR , Minelli C , Abrams KR , Tobin MD , Riley RD . Meta‐analysis of genetic studies using Mendelian randomization—a multivariate approach. Stat Med. 2005;24(14):2241‐2254. 15887296 10.1002/sim.2100

[9] Trikalinos TA , Hoaglin DC , Schmid CH . An empirical comparison of univariate and multivariate meta‐analyses for categorical outcomes. Stat Med. 2014;33(9):1441‐1459. 24285290 10.1002/sim.6044

[10] Berkey CS , Hoaglin DC , Antczak‐Bouckoms A , Mosteller F , Colditz GA . Meta‐analysis of multiple outcomes by regression with random effects. Stat Med. 1998;17(22):2537‐2550. 9839346 10.1002/(sici)1097-0258(19981130)17:22<2537::aid-sim953>3.0.co;2-c

[11] Ishak KJ , Platt RW , Joseph L , Hanley JA . Impact of approximating or ignoring within‐study covariances in multivariate meta‐analyses. Stat Med. 2008;27(5):670‐686. 17492826 10.1002/sim.2913

[12] Jackson D , Riley R , White IR . Multivariate meta‐analysis: potential and promise. Stat Med. 2011;30(20):2481‐2498. 21268052 10.1002/sim.4172 PMC3470931

[13] Riley RD . Multivariate meta‐analysis: the effect of ignoring within‐study correlation. J R Stat Soc A Stat Soc. 2009;172(4):789‐811.

[14] Riley RD , Gates S , Neilson J , Alfirevic Z . Statistical methods can be improved within Cochrane Pregnancy and Childbirth reviews. J Clin Epidemiol. 2011;64(6):608‐618. 21109399 10.1016/j.jclinepi.2010.08.002

[15] Cochrane library: pregnancy and childbirth group, 2017 http://pregnancy.cochrane.org/

[16] Higgins JPT, Green S (editors). Cochrane Handbook for Systematic Reviews of Interventions Version 5.1.0 [updated March 2011]. The Cochrane Collaboration, 2011 Available from http://handbook.cochrane.org.

[17] Cox DR , Snell EJ . Analysis of Binary Data. 2nd ed. New York: Chapman & Hall/CRC; 1989.

[18] DerSimonian R , Laird N . Meta‐analysis in clinical trials. Control Clin Trials. 1986;7(3):177‐188. 3802833 10.1016/0197-2456(86)90046-2

[19] Higgins J , Thompson SG , Spiegelhalter DJ . A re‐evaluation of random‐effects meta‐analysis. J R Stat Soc A Stat Soc. 2009;172(1):137‐159. 10.1111/j.1467-985X.2008.00552.x PMC2667312 19381330

[20] Trikalinos TA , Olkin I . A method for the meta‐analysis of mutually exclusive binary outcomes. Stat Med. 2008;27(21):4279‐4300. 18416445 10.1002/sim.3299

[21] Trikalinos TA , Olkin I . Meta‐analysis of effect sizes reported at multiple time points: A multivariate approach. Clinical Trials. 2012;9(5):610‐620. pmid:22872546 22872546 10.1177/1740774512453218

[22] Wei Y , Higgins J . Estimating within‐study covariances in multivariate meta‐analysis with multiple outcomes. Stat Med. 2013;32(7):1191‐1205. 23208849 10.1002/sim.5679 PMC3618374

[23] White IR . Multivariate random‐effects meta‐analysis. Stata J. 2009;9(1):40‐56.

[24] White IR . Multivariate random‐effects meta‐regression: updates to mvmeta. Stata J. 2011;11(2):255‐270.

[25] Devane D , Lalor JG , Daly S , McGuire W , Smith V . Cardiotocography versus intermittent auscultation of fetal heart on admission to labour ward for assessment of fetal wellbeing. The Cochrane Library. 2012. 10.1002/14651858.CD005122.pub4 22336808

[26] Alfirevic Z , Stampalija T , Roberts D , Jorgensen AL . Cervical stitch (cerclage) for preventing preterm birth in singleton pregnancy. The Cochrane Library. 2012. 10.1002/14651858.CD008991.pub2 22513970

[27] Madden K , Middleton P , Cyna AM , Matthewson M , Jones L . Hypnosis for pain management during labour and childbirth. The Cochrane Library. 2012. 10.1002/14651858.CD009356.pub2 23152275

[28] Derry S , Straube S , Moore RA , Hancock H , Collins SL . Intracutaneous or subcutaneous sterile water injection compared with blinded controls for pain management in labour. The Cochrane Library. 2012. 10.1002/14651858.CD009107.pub2 PMC11663508 22258999

[29] Mackeen AD , Seibel‐Seamon J , Grimes‐Dennis J , Baxter JK , Berghella V . Tocolytics for preterm premature rupture of membranes. The Cochrane Library. 2011. 10.1002/14651858.CD007062.pub2 21975760

[30] Klomp T , van Poppel M , Jones L , Lazet J , Di Nisio M , Lagro‐Janssen AL . Inhaled analgesia for pain management in labour. The Cochrane Library. 2012. 10.1002/14651858.CD009351.pub2 PMC11627147 22972140

[31] Griffiths JD , Gyte GM , Paranjothy S , Brown HC , Broughton HK , Thomas J . Interventions for preventing nausea and vomiting in women undergoing regional anaesthesia for caesarean section. The Cochrane Library. 2012. 10.1002/14651858.CD007579.pub2 PMC4204618 22972112

[32] Frosi G , Riley RD , Williamson PR , Kirkham JJ . Multivariate meta‐analysis helps examine the impact of outcome reporting bias in Cochrane rheumatoid arthritis reviews. J Clin Epidemiol. 2015;68(5):542‐550. 25537265 10.1016/j.jclinepi.2014.11.017

[33] Jackson D , White IR , Price M , Copas J , Riley RD . Borrowing of strength and study weights in multivariate and network meta‐analysis. Stat Methods Med Res. 2015;26(6):2853‐2868. p.0962280215611702 26546254 10.1177/0962280215611702 PMC4964944

[34] Copas JB , Jackson D , White IR , Riley RD . The role of secondary outcomes in multivariate meta‐analysis. J R Stat Soc Ser C Appl Stat. 2018;67(5):1177‐1205. 10.1111/rssc.12274 PMC6193545 30344346

[35] Jackson D , Bujkiewicz S , Law M , Riley R , White I . A matrix‐based method of moments for fitting multivariate network meta‐analysis models with multiple outcomes and random inconsistency effects. Biometrics. 2018;74(2):548‐556. 10.1111/biom.12762 28806485 PMC6038911

[36] Rhodes KM , Turner RM , White IR , Jackson D , Spiegelhalter DJ , Higgins JPT . Implementing informative priors for heterogeneity in meta‐analysis using meta‐regression and pseudo data. Stat Med. 2016;35(29):5495‐5511. 27577523 10.1002/sim.7090 PMC5111594

