# Supplementary material for: Empirical comparison of univariate and multivariate meta‐analyses in Cochrane Pregnancy and Childbirth reviews with multiple binary outcomes
Source: Res Synth Methods. 2019 Aug 12;10(3):440–51. doi: 10.1002/jrsm.1353 (PMC6771837; doi:10.1002/jrsm.1353)
Supplement: Supplementary file 1 — Appendix S1: Univariate random effects model [file JRSM-10-440-s001.docx]

**Appendix 1: Univariate random effects model**

Let $y_{ip}$ be the log-odds ratio estimate for a intervention contrast of interest for outcome p from study $i$. It is assumed that $y_{ip}$ has a normal sampling distribution with true mean $\theta_{ip}$ and standard error $s_{ip}$, the latter of which is assumed known. In the random effects model the studies are allowed to have a different true log-odds ratio drawn from a Normal distribution with overall mean $\theta_{p}$ and between-study variance $\tau_{p}^{2}$ such that:

$$\begin{matrix} y_{ip}\sim N\left(\theta_{ip},s_{ip}^{2}\right) \\ \theta_{ip}\sim N\left(\theta_{p},\tau_{p}^{2}\right) \end{matrix}$$

The model is fit using restricted Maximum Likelihood Estimation using the MVMETA v3.1 command in STATA v14.
