# Supplementary material for: Empirical comparison of univariate and multivariate meta‐analyses in Cochrane Pregnancy and Childbirth reviews with multiple binary outcomes
Source: Res Synth Methods. 2019 Aug 12;10(3):440–51. doi: 10.1002/jrsm.1353 (PMC6771837; doi:10.1002/jrsm.1353)
Supplement: Supplementary file 2 — Appendix S2: Multivariate random effects model [file JRSM-10-440-s002.docx]

**Appendix 2 Multivariate random effects model**

In a standard multivariate random effects meta-analysis we fit a two level hierarchical model. Now let $y_{i}$ denote the vector of estimated log-odds ratios measuring the intervention effect for each outcome in study $i$. The within study estimates are assumed to have a multivariate Normal (MVN) sampling distribution parameterised by the vector of study specific true mean intervention effects $\theta_{i}$, and the estimated within study covariance matrix $S_{i}$, which contains the standard errors and also within-study correlations, each of which is assumed known (See below). The true vector of intervention effects from each study is assumed to be a random draw from a multivariate normal distribution with a vector of mean intervention effects $\theta$, and between study covariance matrix $\Sigma$ , containing P between-study variances which is the number of outcomes and a between-study correlation for each pair of outcomes. The model is written as:

$$\begin{matrix} y_{i}\sim MVN\left(\theta_{i},S_{i}\right) \\ S_{i}=\left[\begin{matrix} s_{i1}^{2} & \rho_{i12}^{\left(w\right)}s_{i1}s_{i2} & \cdots& \rho_{i1P}^{\left(w\right)}s_{i1}s_{iP} \\ . & s_{i2}^{2} & \cdots& \rho_{i2P}^{\left(w\right)}s_{i2}s_{iP} \\ . & . & \ddots& \vdots\\ . & . & . & s_{iP}^{2} \end{matrix}\right] \end{matrix}$$

$$\begin{matrix} \theta_{i}\sim MVN\left(\theta,\Sigma\right) \\ \Sigma=\left[\begin{matrix} \tau_{1}^{2} & \rho_{12}^{\left(b\right)}\tau_{1}\tau_{2} & \cdots& \rho_{1P}^{\left(b\right)}\tau_{1}\tau_{P} \\ . & \tau_{2}^{2} & \cdots& \rho_{2P}^{\left(b\right)}\tau_{2}\tau_{P} \\ . & . & \ddots& \vdots\\ . & . & . & \tau_{P}^{2} \end{matrix}\right] \end{matrix}$$

where $\rho_{iqp}^{\left(w\right)}$ is the estimated study-specific within-study correlation coefficient between $y_{i,q}$ and $y_{i,p}$, and$\rho_{qp}^{\left(b\right)}$is the estimated between study correlation coefficient between the estimated means $\hat{\theta}_{q}$ and $\hat{\theta}_{p}$.
