# Supplementary material for: Empirical comparison of univariate and multivariate meta‐analyses in Cochrane Pregnancy and Childbirth reviews with multiple binary outcomes
Source: Res Synth Methods. 2019 Aug 12;10(3):440–51. doi: 10.1002/jrsm.1353 (PMC6771837; doi:10.1002/jrsm.1353)
Supplement: Supplementary file 3 — Appendix S3: Riley overall correlation model [file JRSM-10-440-s003.docx]

**Appendix 3: Riley overall correlation model**

The model described above is fully hierarchical in that the within-study covariance matrices $S_{i}$ are fully partitioned from the between study covariance matrix $\Sigma$. An alternative, partial hierarchical model has been proposed by Riley et al [3] in which the within-and between study correlations are subsumed into a single parameter which represents the overall correlation. The full model is written as follows:

$$y_{i}\sim MN\left(\theta,\Phi_{i}\right)$$

$$\Phi_{i}=\left(\begin{matrix} \psi_{1}+s_{i,1}^{2} & \rho_{12}\sqrt{\left(\psi_{1}+s_{i,1}^{2}\right)\left(\psi_{2}+s_{i,2}^{2}\right)} & \cdots& \rho_{1p}\sqrt{\left(\psi_{1}+s_{i,1}^{2}\right)\left(\psi_{p}+s_{i,P}^{2}\right)} \\ . & \psi_{2}+s_{i,2}^{2} & \cdots& \rho_{2p}\sqrt{\left(\psi_{2}+s_{i,2}^{2}\right)\left(\psi_{p}+s_{i,P}^{2}\right)} \\ . & . & \ddots& \vdots\\ . & . & . & \psi_{p}+s_{i,P}^{2} \end{matrix}\right)$$

In this model $\psi_{p}$ is the additional between-study variability beyond sampling error. $s_{i,p}^{2}$ are the study and outcome specific variances of the log-odds ratios, and $\rho_{qp}$is the overall within and between studies correlation between outcomes $q$ and $p$. Further details of the properties of this model can be found in Riley [3].
