# Supplementary material for: Empirical comparison of univariate and multivariate meta‐analyses in Cochrane Pregnancy and Childbirth reviews with multiple binary outcomes
Source: Res Synth Methods. 2019 Aug 12;10(3):440–51. doi: 10.1002/jrsm.1353 (PMC6771837; doi:10.1002/jrsm.1353)
Supplement: Supplementary file 4 — Appendix S4: Computational methods [file JRSM-10-440-s004.docx]

**Appendix 4: Computational methods**

Several computational methods were used to fit the Riley overall correlation model:

1. STATA v14’s default REML algorithm via the MVMETA v3.1 command.
2. The REML algorithm using a range of starting values for the overall variance matrix.
3. Using the Broyden-Fletcher-Goldfarb-Shanno (BFGS) algorithm

The model was deemed not to have converged after 2,000 iterations. Further checks were applied to those models which converged. The Riley model is known to be unstable when very high (positive or negative) values for the overall correlation are estimated so any results where the correlations were > 0.95 or < -0.95 are rejected [3]. The models were refit assuming the overall correlation matrix was fixed and known with values set equal to the estimates from the BGFS algorithm. Standard errors for the effect estimates using the BGFS method were compared to these and models were rejected if the standard error was higher when the overall correlation matrix was assumed fixed and known.
